# Right atrial presssure and intra-abdominal pressure: the elephant in the room

**DOI:** 10.1186/s13613-025-01478-4

**Published:** 2025-05-09

**Authors:** Ngan Hoang Kim Trieu

**Affiliations:** Department of Intensive Care Medicine, Cho Ray Hospital, 201B Nguyen Chi Thanh Street, District 5, Ho Chi Minh City, Vietnam

Dear Editor,

I read with great interest the Letter to the Editor by Magder [1], recently published in *Annals of Intensive Care*, which enhances the understanding of right atrial pressure (RAP) in the application of Guyton’s approach to altering cardiac output for clinical fluid management, building upon the author’s prior publication [2]. The letter provides an in-depth exploration, emphasizing the pivotal role of right atrial pressure (RAP) in guiding fluid therapy. I appreciate this thorough discussion, as it highlights several nuanced insights into hemodynamic assessment in critically ill patients. 

In the 1950s, Arthur Guyton argued that “*cardiac output is determined by the interaction of two functions: (1) a function that determines the return of blood from the peripheral circulation*,* that is*,* venous return, and (2) a function that determines the output from the heart acting as a pump”.* The human body functions as a cohesive system, and it is inappropriate to separate it into isolated components based on a simplified model, particularly in critically ill patients. However, despite the comprehensive insights provided by Magder, the impact of intra-abdominal pressure (IAP) on RAP and hemodynamics remains underexplored.

## Why is intra-abdominal pressure discussed here?

Current studies have reported that the incidence of intra-abdominal hypertension ranges from 30 to 50% in critically ill patients —an alarming number [3]. Building on this foundation, I would like to draw attention to the often-overlooked challenge of IAP on RAP and its broader hemodynamic consequences. It should be noted that IAP affects cardiac function by altering RAP and venous return. Specifically, IAP directly impacts RAP through multiple mechanisms:

1. *Impedance of venous return*: Increased IAP compresses the inferior vena cava and abdominal vasculature, thereby reducing venous return to the heart. This can result in a paradoxical rise in RAP without a corresponding improvement in cardiac output.
2. *Increased intrathoracic pressure*: Elevated IAP raises intrathoracic pressure, decreasing left ventricular compliance and increasing right ventricular afterload through ventricular interdependence. This contributes to both transmural and intramural RAP elevation, complicating the interpretation of hemodynamic changes.
3. *Compromised organ perfusion*: Increased IAP also reduces systemic organ perfusion and increases systemic afterload, further impairing cardiac function and perfusion dynamics.

As pointed out by Magder, “*The normal range of RAP is small*,* and measurements must be done accurately”* [1]. However, when IAP is elevated, RAP values can become misleading if not interpreted in the appropriate clinical context. Given the linear correlation between IAP and RAP, incorporating IAP measurement into hemodynamic assessment offers a more comprehensive understanding of a patient’s status [4, 5]. The clinical impact of IAP on RAP is often underappreciated. Clinicians should integrate IAP measurement into hemodynamic monitoring, especially in patients with risk factors such as sepsis, burn, abdominal trauma, or other intra-abdominal pathology to better evaluate and make decisions in optimizing fluid management.

In conclusion, Magder’s letter effectively clarifies the application of Guyton’s hemodynamic model and highlights its simplicity and practicality for clinical fluid management. However, its application requires careful interpretation, as patients represent a complex interplay of multiple interacting organ systems. Hemodynamic evaluation should include “non-hemodynamic parameters” with IAP considered an essential factor. Further research is necessary to explore the incorporation of IAP into hemodynamic parameters and to individualize its evaluation across various populations.

## Data Availability

Not applicable.
